# Participación femenina en los comités editoriales de revistas médicas en Latinoamérica

**DOI:** 10.7705/biomedica.6120

**Published:** 2022-06-01

**Authors:** Christian Renzo Aquino-Canchari, Sarai Gloria Chávez-Bustamante, Christeam A. Benites-Ibarra, Renatta Quijano-Escate, Hugo Arroyo-Hernández

**Affiliations:** 1 Universidad Peruana Los Andes, Sociedad Científica de Estudiantes de Medicina Los Andes (SOCIEMLA), Huancayo, Perú Universidad Peruana Los Andes Universidad Peruana Los Andes Sociedad Científica de Estudiantes de Medicina Los Andes (SOCIEMLA) Huancayo Peru; 2 Universidad Continental, Sociedad Científica Médico Estudiantil Continental (SOCIMEC), Huancayo, Perú Universidad Continental Sociedad Científica Médico Estudiantil Continental (SOCIMEC) Huancayo Perú; 3 Universidad Nacional del Santa, Sociedad Científica de Estudiantes de Medicina de la Universidad Nacional del Santa (SOCEMUNS), Nuevo Chimbote, Perú Universidad Nacional del Santa Universidad Nacional del Santa Sociedad Científica de Estudiantes de Medicina de la Universidad Nacional del Santa (SOCEMUNS) Nuevo Chimbote Peru; 4 Universidad Nacional San Luis Gonzaga, Sociedad Científica de Estudiantes de Medicina de Ica (SOCEMI), Ica, Perú Universidad Nacional San Luis Gonzaga Universidad Nacional San Luis Gonzaga Sociedad Científica de Estudiantes de Medicina de Ica (SOCEMI) Ica Peru; 5 Oficina General de Información y Sistemas, Instituto Nacional de Salud, Lima, Perú Oficina General de Información y Sistemas Instituto Nacional de Salud Lima Perú

**Keywords:** publicaciones periódicas como asunto, bibliometría, editorial, equidad de género, América Latina, Periodicals as topic, bibliometrics, editorial, gender equity, Latin America

## Abstract

**Introducción.:**

La participación femenina en el campo de la medicina y la investigación se ha incrementado en los últimos años; sin embargo, aún existen inequidades en la proporción de hombres y mujeres, especialmente en los cargos directivos y la participación en los comités editoriales de revistas científicas.

**Objetivo.:**

Establecer la participación femenina en los comités editoriales de revistas médicas en Latinoamérica, y explorar su asociación con los cargos editoriales y los indicadores de impacto.

**Materiales y métodos.:**

Se hizo un estudio descriptivo de tipo bibliométrico de revistas médicas de Latinoamérica indizadas en Scopus, actualizadas y vigentes en el 2020, las cuales se seleccionaron del portal de *Scimago Journal & Country Rank*. Los equipos editoriales se categorizaron en tres grupos según sus funciones y, posteriormente, se registró el sexo de sus miembros a partir de sus nombres.

**Resultados.:**

Se incluyeron 113 revistas. En cuanto al liderazgo editorial, entre los 264 directores de comités editoriales, las mujeres representaban el 12,9 %. En lo concerniente a las diferentes funciones, de 1.449 miembros, las mujeres eran el 28,9 %, en tanto que, de los 4.575 miembros de comités consultivos, el 19,0 % correspondía a mujeres. Se observó una mayor presencia de mujeres en los comités editoriales de revistas de Chile, Brasil y Venezuela, y en las especialidades de salud pública, pediatría y anestesiología.

**Conclusiones.:**

La participación femenina en los comités editoriales de revistas médicas de Latinoamérica es escasa.

## Abstract (en)

**Introduction:**

Female participation in the field of medicine and research has increased in recent years; however, there are still inequities in the proportion of men and women in medical leadership, especially in management positions and editorial committees of scientific journals.

**Objective:**

To identify female participation in the editorial committees of medical journals in Latin America and explore the association with editorial positions and impact indicators.

**Materials and methods:**

We conducted a descriptive bibliometric study to determine female participation in the editorial committees of medical journals in Latin America. We included 113 medical journals published in Latin America and indexed in Scopus, updated and current in 2020, selected from the Scimago Journal & Country Rank portal. The gender of editorial committee members was identified on the web pages of each magazine.

**Results:**

Regarding editorial leadership in the 113 journals included, women represented 12.9% of 264 members; as for the functions within the editorial committee, of 1,449 members, 28.9% were women while in advisory committees, of 4,575 members 19.0% were women. The presence of women in editorial committees was higher in journals from Chile, Brazil, and Venezuela in specialties such as public health, pediatrics, and anesthesiology.

**Conclusions:**

Female participation in the editorial committees of medical journals in Latin America is low.

Tradicionalmente, las oportunidades brindadas a varones y mujeres han sido desiguales. Para contrarrestar dicha tendencia, en este siglo la equidad de género se considera un factor determinante de la salud ^1^. El Índice Global de Brecha de Género, el cual evalúa a 153 países en su progreso hacia la igualdad de género en salud, educación, economía y política, arroja una brecha actual del 31,4 % a nivel mundial, situando a la región de Latinoamérica y el Caribe en el tercer lugar entre ocho regiones analizadas ^2^.

La inequidad de género repercute en el desarrollo profesional de las mujeres ^3^^,^^4^. Está comprobado que las profesionales reciben hasta la mitad del sueldo que sus pares varones, lo que se justifica aduciendo el nivel de especialización y de producción científica ^5^^,^^6^. También, en el financiamiento de los proyectos de investigación hay inequidad, con una clara ventaja para los varones ^7^, es decir, la distribución de los recursos y las oportunidades entre varones y mujeres sigue siendo desigual.

Los comités editoriales de las revistas científicas están conformados por equipos de profesionales cuyas funciones incluyen la gestión de los procesos de publicación de los manuscritos y su evaluación. La participación de las profesionales en estos equipos ha venido aumentando paulatinamente en las últimas cuatro décadas; sin embargo, sigue siendo poca ^8^. El porcentaje de participación varía según la especialidad de la revista: se reporta 3,8 % de presencia en revistas de cirugía ortopédica ^9^; 13,3 % en revistas de anestesia cardiotorácica ^10^ y 23,1 % en revistas de radiología general ^11^.

Hay datos sobre la participación de las mujeres en revistas médicas de Norteamérica, Europa y Asia ^12^^,^^13^, y un breve reporte sobre revistas médicas en Perú ^14^, pero no hay un registro a nivel latinoamericano. Por ello, el objetivo del presente estudio fue determinar la participación femenina en los comités editoriales de revistas médicas en Latinoamérica.

## Materiales y métodos

### Diseño del estudio

Se hizo un estudio transversal y descriptivo de tipo bibliométrico, en el que se incluyeron revistas médicas editadas en Latinoamérica e indizadas en Scopus. Se seleccionaron como mínimo dos revistas de cada especialidad médica con publicación actualizada para el 2020, en cuyas páginas web apareciera la composición del comité editorial.

### Procedimientos

Se realizó una búsqueda en la plataforma de *SCImago Journal and Country Rank* (SJR) (https://www.scimagojr.com) de todos los títulos de revistas en el área de “medicina”, en “todas las categorías” y para la región de “América Latina”. Posteriormente, se accedió a las páginas web oficiales de cada revista y se ingresaron en una base de datos los nombres de los integrantes de sus comités editoriales. La selección se hizo entre el 11 y el 19 de junio del 2020.

### Variables

La variable de género se categorizó en hombres y mujeres a partir de los nombres de pila; en caso de dudas sobre la identidad del sexo, se ingresaban los nombres y apellidos en el motor de búsqueda de Google (Google Inc., Mountain View, Palo Alto, California, USA) para determinarla con base en las imágenes de los perfiles publicados en las páginas web de instituciones académicas, científicas o gubernamentales, así como en redes sociales para profesionales (LinkedIn, ResearchGate, Academia.edu) o generales (Facebook, Twitter, Instagram) de acceso libre.

También, se estableció la variable de la conformación de los equipos editoriales categorizada según las funciones de sus miembros, en tres grupos: el de liderazgo editorial, integrado por directores(as) o editores(as) en jefe, es decir, los cargos editoriales principales y de mayor prestigio científico en la revista; el correspondiente a los comités editoriales integrados por editores(as), incluidos los editores asociado(as) o adjuntos(as) encargados de los procesos editoriales de los manuscritos, y el del comité consultivo, conformado por profesionales de reconocida experticia con funciones consultivas, de revisión y de asesoría en caso de dudas editoriales.

Se evaluaron, además, las variables de especialidad médica de las revistas según las categorías del *SCImago Journal and Country Rank* (SJR), y las revistas médicas no especializadas se consideraron como misceláneas. Se contemplaron, también, el país de edición de la revista y su especialidad, así como la clasificación del *SCImago Journal and Country Rank* (SJR) por cuartiles (Q), donde Q1 corresponde al valor más alto de influencia científica por el número de citas y el prestigio de las revistas donde aparecen. Se consideró, asimismo, el índice de impacto del *SCImago Journal and Country Rank* (SJR) ^15^^,^^16^.

### Análisis estadístico

Se hicieron análisis de frecuencias relativas y absolutas según las funciones de las mujeres en los comités editoriales y las características de las revistas. Para las variables numéricas, se determinó la mediana con su rango intercuartílico (RIC), previa comprobación de la normalidad mediante la prueba de Shapiro Wilk. La prueba de Kruskall-Wallis se utilizó para determinar si la presencia de mujeres en puestos de liderazgo editorial, o según el cuartil de la revista, presentaba diferencias en cuanto a su número en el comité editorial y el comité consultivo. Además, se evaluó si los puestos editoriales según género presentaban diferencias en los indicadores del *SCImago Journal and Country Rank*. Se consideró como estadísticamente significativo un valor de p<0,05. Los análisis se hicieron con el programa estadístico Stata™, versión 22.0.

### Consideraciones éticas

El estudio no requirió la aprobación de un comité de ética institucional, ya que los datos se encuentran disponibles públicamente y son de acceso libre.

## Resultados

**Figura 1:**
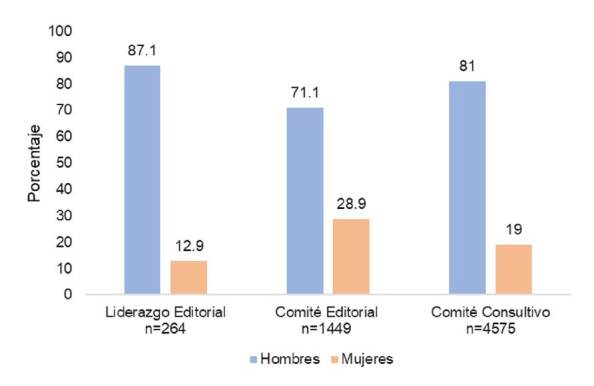
Distribución por género, según funciones editoriales en revistas médicas de Latinoamérica indizadas en Scopus, 2019

De las 129 revistas médicas latinoamericanas indizadas en Scopus durante el 2020, se excluyeron 16. En las 113 incluidas, de 6.448 profesionales participantes, el 21,2 % correspondía a mujeres. En cuanto a los cargos de liderazgo editorial, de 264 miembros, las mujeres representaban el 12,9 %; de los 1.449 miembros de los comités editoriales, el 28,9 % era de sexo femenino y en el grupo de los comités consultivos, de 4.575 miembros, el 19,0 % correspondía a mujeres (figura 1).

**Figura 2:**
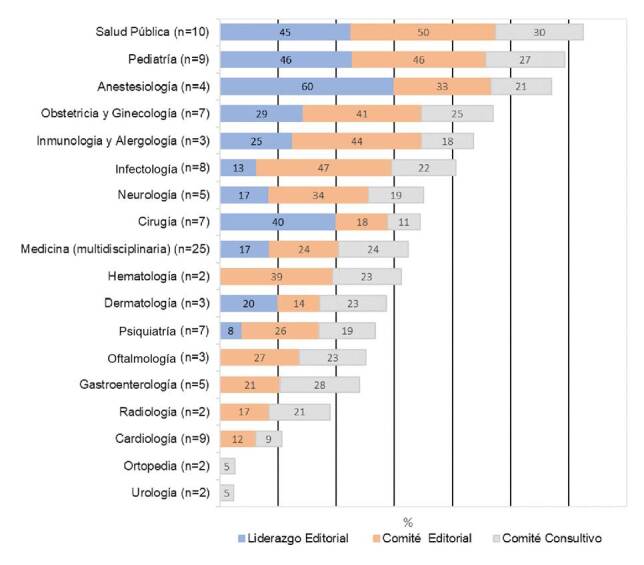
Proporción de funciones editoriales con participación femenina según especialidades de revistas médicas de Latinoamérica indizadas en Scopus, 2019

Entre las 18 especialidades de las revistas médicas analizadas, hubo una mayor presencia de mujeres en las de pediatría y anestesiología; en estas revistas también se encontró una participación igual o superior al 45 % de mujeres en puestos de liderazgo editorial. Por otra parte, en las revistas de hematología, oftalmología, gastroenterología, radiología, cardiología, ortopedia y urología, no se registró la participación de mujeres en puestos de liderazgo editorial. En las revistas de enfermedades infecciosas y pediatría, también se registró una proporción de 45 % o más de mujeres en los cargos de los comités editoriales, en tanto que no hubo participación de mujeres en las revistas de ortopedia y urología. Globalmente, en todas las especialidades, la participación de las mujeres en los comités consultivos fue menor o igual al 30 % (figura 2).

**Figura 3:**
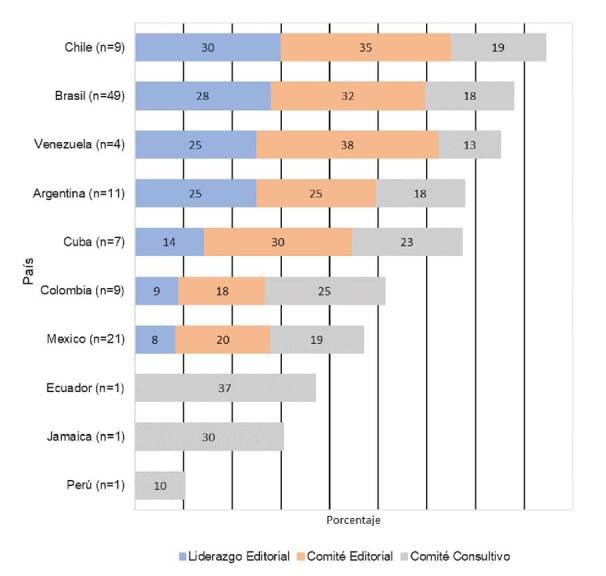
Proporción de funciones editoriales con participación femenina según países en revistas médicas de Latinoamérica indizadas en Scopus, 2019

En cuanto al liderazgo editorial, la presencia de mujeres llegó como máximo a un 25 a 30 % en revistas de Chile, Brasil, Venezuela y Argentina; en los comités editoriales su presencia llegó a un máximo de 25 a 38 % en revistas de Venezuela, Brasil, Cuba y Argentina, en tanto que no se encontraron mujeres en puestos de liderazgo editorial en revistas de Ecuador, Jamaica y Perú, pero la presencia de mujeres como parte de los comités consultivos fue mayor en Ecuador (37%) y Jamaica (30%) (figura 3).

La presencia de mujeres en cargos de liderazgo editorial se asoció con su mayor número en los comités editoriales (mediana de 6 mujeres, RIC: 2-6; p=0,001), en comparación con los cargos de liderazgo editorial conformados solo por hombres. Además, se encontró una asociación entre las revistas clasificadas en Q1 y Q2 y un mayor número de mujeres en los comités editoriales (mediana 6, RIC: 2-15; p=0,007); estas diferencias no se encontraron en los comités consultivos (cuadro 1).

**Cuadro 1:** Mujeres en puestos de liderazgo editorial y cuartil de la revista y su relación con la presencia de mujeres en puestos de comité editorial y consultivo en revistas médicas de Latinoamérica indizadas en Scopus, 2019

	Mujeres en el comité editorial					Mujeres en el comité consultivo				
	Revistas	%*	Mediana de mujeres	RIC	p	Revistas	%*	Mediana de mujeres	RIC	p
Mujeres en liderazgo editorial										
No	53/79	67,1	2	(0 a 3)	0,001	71/76	93,4	6	(3 a 12)	0,269
Sí	22/27	81,5	6	(2 a 6)		23/24	95,8	9	(3 a 16)	
Cuartil										
Q3 - Q4	62/91	68,1	2	(0 ta 4)	0,007	81/87	93,1	7	(3 a 13)	0,746
Q1 - Q2	13/15	86,7	6	(2 a 15)		13/13	100	4	(2 a 16)	

RIC: rango intercuartílico

*Proporción de revistas con presencia de mujeres en el comité editorial o consultivo según cada categoría

Asimismo, se encontró una asociación entre la participación de mujeres en puestos de liderazgo editorial y un mejor indicador en el SJR (SJR: 0,223; RIC: 0,139 - 0,481; p=0,031) comparado con puestos de liderazgo editorial exclusivamente en manos de hombres; no se encontraron diferencias en el indicador del SJR con la participación o ausencia de mujeres en los comités editoriales (p=0,102) y consultivos (p=0,766) (cuadro 2).

**Cuadro 2:** Participación de mujeres según puestos editoriales y su relación con el indicador de *SCImago Journal and Country Rank* en revistas médicas de Latinoamérica indizadas en Scopus, 2019

Participación de mujeres		Mediana del SJR	RIC	p
Liderazgo editorial				
	No	0,169	(0,122 a 0,269)	0,031
	Sí	0,223	(0,139 a 0,481)	
Comité editorial				
	No	0,156	(0,121 a 0,268)	0,102
	Sí	0,211	(0,132 a 0,389)	
Comité consultivo				
	No	0,173	(0,128 a 0,370)	0,766
	Sí	0,187	(0,162 a 0,239)	

RIC: rango intercuartílico

## Discusión

Los hallazgos del estudio muestran que las funciones editoriales en las revistas médicas de Latinoamérica indizadas en Scopus están a cargo principalmente de hombres. Sin embargo, en áreas como la salud pública, la pediatría y la anestesiología, así como en las revistas de Chile, Brasil y Venezuela, hubo mayor participación femenina. Asimismo, la presencia de mujeres en puestos de liderazgo editorial se asoció con un mayor número de mujeres en los comités editoriales; además, las revistas con mejores cuartiles de impacto y un mejor indicador de citación, tenían una mayor presencia de mujeres en los comités editoriales y en cargos de liderazgo editorial.

Nuestro estudio demuestra que las mujeres son una minoría en las diversas funciones editoriales y están ausentes de los cargos de liderazgo editorial en algunas especialidades y países, lo que se ha reportado previamente en otros estudios ^17^^-^^19^. La participación en los comités editoriales requiere un perfil profesional con experiencia científica, algo que las mujeres que inician una carrera en la investigación no logran del todo, pues tienen un mayor riesgo de deserción que su contraparte masculina ^20^, como queda demostrado con su menor presencia en las carreras de ciencia, tecnología, ingeniería y matemática, incluso hoy, cuando logran grados académicos y títulos de posgrado en número similar a los varones ^21^.

La selección para los cargos editoriales se hace entre quienes tienen mayores niveles académicos y ostentan cargos directivos en órganos de gobierno de universidades o instituciones científicas, por ello, el que haya menos mujeres con estas características explicaría esta desigualdad en la conformación de los comités editoriales ^22^. A esto se debe sumar el histórico déficit de mujeres en algunas especialidades como urología y ortopedia ^9^, aunque en las últimas décadas se ha comenzado a ver una tendencia creciente de participación femenina en otras especialidades como la anestesiología ^10^.

La baja representatividad de mujeres en los comités editoriales es notoria en los países de Latinoamérica, donde la participación de los hombres representa más del doble que la de las mujeres. Al comparar este hallazgo con el actual índice global de brecha de género, países como Cuba y Ecuador mostraron un gran progreso en su reducción ^2^, situación que no se ve reflejada en el presente estudio, pues en ambos países se evidenció una baja participación de mujeres en los comités editoriales de sus revistas.

Lograr la igualdad de género en la composición editorial es complejo, pues se trata de un problema de múltiples factores en un campo dominado históricamente por hombres y con poco recambio en los cargos de liderazgo editorial ^5^^,^^23^^,^^24^ dada la mayor carga social y de obligaciones familiares de las mujeres en comparación con los hombres, lo que se cuenta entre las principales limitaciones para dedicar tiempo adicional a las labores editoriales ^11^^,^^12^^,^^25^. Las políticas nacionales de incentivos para las investigadoras aún son limitadas ^26^, sin embargo, se ha evidenciado que en países donde la brecha de género es menor, las mujeres que cursan carreras STEM son pocas, pese a tener más oportunidades educativas y de empoderamiento ^27^. Una buena propuesta es la que han adoptado algunas revistas médicas internacionales de alcanzar la paridad de género en los comités editoriales como un signo visible de progreso continuo que sirva de ejemplo a las mujeres jóvenes que contemplan una carrera científica ^28^^-^^30^.

La presencia de mujeres en puestos de liderazgo editorial se asoció con su mayor número en los comités editoriales, mas no así en los consultivos, lo que indicaría una gestión editorial en aquel nivel más cercana y colaborativa entre mujeres. Este hallazgo es similar al de un análisis de 69 revistas de diversas especialidades odontológicas ^31^, pero difiere de lo reportado en otro de revistas de psiquiatría en el que las mujeres en puestos de liderazgo editorial se asoció a su menor presencia en los comités editoriales y consultivos ^19^. Por lo tanto, no podrían hacerse inferencias con respecto a otras revistas especializadas o, incluso, países.

Asimismo, la ubicación de las revistas médicas en un mejor cuartil (Q1-Q2) se asoció con la presencia de más mujeres en los comités editoriales, en tanto que su presencia en puestos de liderazgo editorial se asoció con mejores indicadores de citación (SJR), pues, si bien son pocas las mujeres que integran estos cargos editoriales, requieren de una mayor experiencia en la publicación científica, hecho que se ha reportado en otros estudios en los que las revistas con mayor factor de impacto parecen favorecer la inclusión de mujeres ^19^^,^^32^^,^^33^. La presencia de mujeres en revistas de impacto en una especialidad o país podría generar modelos y convertirlas en mentoras que, por medio del establecimiento de redes de información y apoyo, contribuyan a incrementar las posibilidades de promoción de las mujeres en el ámbito de las revistas científicas.

Nuestro estudio tiene algunas limitaciones. Se consideraron únicamente las revistas médicas latinoamericanas incluidas en el SJR; sin embargo, al ser Scopus una base de datos internacional, se pudo sistematizar la recolección de los datos sin olvidar que varias de las revistas seleccionadas se encontraban en otras bases de datos internacionales y regionales. Otra limitación es que la estructura organizativa de algunas revistas podría contemplar roles en sus comités editoriales diferentes a los analizados aquí, y que sus páginas web podían estar desactualizadas en el momento de la recolección de los datos. Además, aunque muchas revistas médicas siguen estándares internacionales para la conformación de sus comités editoriales, podrían verse influenciadas por aspectos económicos, administrativos o de política institucional.

Por último, los hallazgos deben generalizarse con cautela dada la temporalidad y la capacidad limitadas de los diseños transversales, así como la disparidad en los números, lo que dificulta hacer inferencias. Además, no puede afirmarse que exista un sesgo de género en las revistas, ignorando que este también existe en la productividad científica, como se ha señalado en estudios previos.

En conclusión, la participación de las mujeres en los cargos editoriales de las revistas médicas de Latinoamérica es escasa. Las investigadoras tienen una mayor participación en aquellas revistas ubicadas en un mejor cuartil de impacto. Podrían adoptarse estrategias editoriales que busquen una participación más inclusiva de las mujeres y, por ende, una mayor diversidad de perspectivas de investigación y colaboración.
